# Computed Tomography-Based Morphometric Analysis of the Ascending Aorta in Acute Type A Dissection Beyond Diameter-Based Thresholds: A National Cohort Study from Latvia

**DOI:** 10.3390/medsci14020204

**Published:** 2026-04-19

**Authors:** Ivars Brecs, Sandra Skuja, Simons Svirskis, Nityanand Jain, Peteris Stradins

**Affiliations:** 1 Centre of Cardiac Surgery, Pauls Stradinš Clinical University Hospital, 13 Pilsonu Street, LV-1002 Riga, Latvia; 2 Joint Laboratory of Electron Microscopy, Riga Stradinš University, 9 Kronvalda Boulevard, LV-1010 Riga, Latvia; 3 Institute of Microbiology and Virology, Riga Stradinš University, 5 Ratsupites Street, LV-1067 Riga, Latvia; 4 Independent Statistical Consultant, Chandigarh Capital Region, Chandigarh 160036, India

**Keywords:** thoracic aorta, aortic dissection, acute type A aortic dissection, ascending aortic diameter, ascending aortic length, aortic size index

## Abstract

**Background/Objectives**: Ascending aortic aneurysm is a heterogeneous disease, with many cases of acute Stanford type A aortic dissection (ATAAD) presenting with aortic diameters below currently recommended surgical thresholds. Demographic factors such as age and sex, along with indexed aortic size groups, have been proposed to improve risk stratification. **Methods**: We included 65 adult patients who underwent surgical intervention for ATAAD. Morphometric measurements were obtained from computed tomography angiography (CTA) using centerline reconstruction. Maximum ascending aortic diameter and length were measured. Indexed parameters included the aortic size index (ASI), aortic height index (AHI), aortic length index (ALI) and cross-sectional aortic area indexed to height (CSA/H). Estimated pre-dissection dimensions were derived by reducing diameter by 18% and length by 2.7%. The cohort was stratified by age-, sex-, and ASI-defined groups. **Results**: Women were older than men (mean age 67 [SD 11] vs. 58 [SD 13] years, *p* = 0.01). Aortic diameter and length did not differ significantly by age or sex. At presentation, an ascending aortic diameter < 5.0 cm was observed in 37.1% of patients aged < 65 years and 26.7% of those aged ≥ 65 years. When stratified by sex, 25.0% of women and 35.6% of men presented with an ascending aortic diameter < 5.0 cm. Indexed parameters (ALI, AHI and ASI) were higher in older patients and women despite their smaller body size. In estimated pre-dissection analyses, less than 10% of the patients had diameters ≥ 5.5 cm, whereas most had estimated diameters < 5.0 cm. **Conclusions**: A substantial proportion of patients with ATAAD present with aortic dimensions below the current surgical thresholds. These findings underscore the limitations of diameter-based criteria and support the potential value of indexed geometric parameters in improving risk assessment in ATAAD patients.

## 1. Introduction

Ascending aortic aneurysm (AsAA) is a heterogeneous disease characterized by marked demographic variation and significant clinical impact, highlighting the need for more personalized and tailored approaches to risk stratification [1]. This heterogeneity is well demonstrated by data from a Danish population-based study in which ascending aortic dilatation was observed in 4.0% of men and 2.1% of women, whereas AsAA was documented in only 0.1% of both sexes [2]. Sex-related differences in aortic dimensions have also been described in patients presenting with acute type A aortic dissection [3].

While diameter-based thresholds remain central to surgical decision-making, a substantial proportion of patients with acute Stanford type A aortic dissection (ATAAD) present with aortic diameters below established operative criteria. Data from the International Registry of Acute Aortic Dissection (IRAD) indicated that more than half of the patients with acute type A dissection have ascending aortic diameters below the recommended surgical thresholds [4]. Such analyses are complicated by the fact that the measurements obtained at presentation may not always accurately reflect the pre-dissection aortic diameter, as the dissection process itself can alter aortic dimensions [5,6]. Consequently, reliance on diameter thresholds alone may fail to identify a considerable subset of pre-dissection phase patients who are at risk yet remain below the intervention threshold for prophylactic ascending aortic repair [7,8].

To address these limitations, additional geometric parameters have been explored to improve risk assessment in ascending aortic disease. Among these, ascending aortic (AsA) length has emerged as a potential complementary morphometric marker. Computed tomography angiography (CTA) based studies have shown that patients with acute type A dissection have significantly greater AsA length compared with both controls and patients with stable aneurysms, independent of age, sex, and aortic diameter [9]. Notably, AsA length appears to be less affected by acute geometric distortion during dissection, suggesting that measurements obtained at presentation may better approximate pre-dissection aortic geometry than diameter alone [5]. Building on these findings, several derived geometric indices have recently been proposed to further refine individual risk stratification in patients with ascending aortic disease [10,11,12].

Hence, in the present study we evaluated aortic geometric parameters measured at presentation and estimated pre-dissection values, including body size-indexed measures, in patients treated for ATAAD. Morphometric findings were compared across subgroups defined by age, sex, and aortic size index (ASI) to gain further exploratory insights.

## 2. Methods

The present study was approved by the Ethics Committee of Riga Stradinš University (Decision No. 4/dated 28 June 2018) and conducted according to the principles described in the Declaration of Helsinki. Written informed consent was obtained from all study participants.

### 2.1. Study Population

This retrospective study included consecutively treated adult patients aged > 18 years who underwent surgical intervention for ATAAD at the Surgical Clinic at Pauls Stradinš Clinical University Hospital (PSCUH) between January 2019 and December 2023. As PSCUH is the only centre in Latvia providing cardiac surgery, the study cohort represented all surgically treated patients with ATAAD nationwide during the study period.

### 2.2. Surgical Indications

Indications for surgical intervention were determined according to the 2014 European Society of Cardiology (ESC) Guidelines on the Diagnosis and Treatment of Aortic Diseases [13]. All patients fulfilled Class I indications for urgent surgical treatment (level of evidence B). In brief, a Class I indication denotes a strong recommendation for surgical intervention. In this context, acute type A aortic dissection is regarded as a Class I indication, particularly when accompanied by life-threatening complications such as malperfusion, aortic valve involvement, or pericardial tamponade.

### 2.3. Exclusion Criteria

Patients were excluded from the study if any of the following were present: (i) missing and/or inadequate quality of preoperative computed tomography angiography (CTA) imaging records; (ii) penetrating aortic ulcer or intramural hematoma without dissection; (iii) iatrogenic or traumatic aortic dissection, or a history of type B aortic dissection; and/or (iv) known connective tissue disease.

### 2.4. Patient Characteristics

Demographic and anthropometric data were obtained from electronic medical records, including sex, age, height, and weight. Aortic valve morphology was classified as bicuspid or tricuspid. Anthropometric variables were collected primarily to derive and interpret body-size-indexed aortic measures. Although these variables were also explored in descriptive and correlation analyses, they were not considered as independent predictors for clinical decision-making. To gain structured insights into morphometric parameters across clinically relevant groups, we stratified our cohort based on age (<65 and ≥65 years) [14] and sex (male and female) [15].

### 2.5. Imaging Analysis and Morphometric Measurements

All morphometric analyses were performed using contrast-enhanced CTA acquired at the time of ATAAD and analyzed with GE Healthcare AW Server (v3.2 EXT 6.0; GE Healthcare, Chicago, IL, USA). A semi-automated centerline of the entire thoracic aorta was generated and manually adjusted to ensure anatomical alignment. All measurements were obtained in planes perpendicular to the blood flow based on the centerline reconstruction [9,16]. Measurements were obtained at reproducible anatomical landmarks and at the level of maximal ascending aortic dimension, consistent with standard morphometric assessment of ascending aortic geometry.

**Figure 1:**
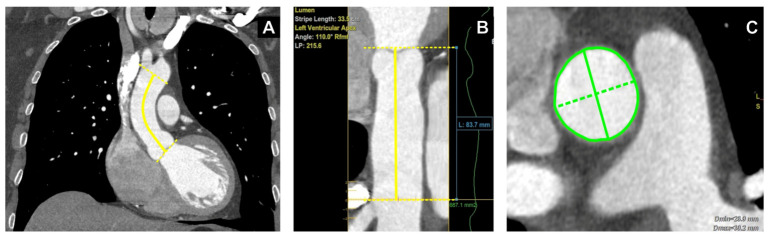
Computed tomography angiography (CTA) based morphometric assessment of the ascending aorta. (**A**) Coronal CTA overview of the thoracic aorta showing the analyzed segment of the ascending aorta. The solid yellow curved line indicates the aortic centerline while the dashed yellow transverse lines at the ends of the solid line indicate the proximal and distal landmarks used for ascending aortic length assessment. Ascending aortic length was measured along the centerline from the aortic annulus to the origin of the innominate artery. (**B**) Reconstructed view of the assessed segment showing centerline-based measurement of ascending aortic length. The solid yellow vertical line indicates the measured ascending aortic length, and the yellow dashed transverse lines indicate the reference levels defining the measured segment. (**C**) Cross-sectional CTA image at the level of the maximal ascending aortic dimension. The solid green line represents the maximal diameter, and the green dashed line represents the perpendicular short-axis diameter. The maximal diameter, measured as the distance between two oppositely positioned points along the circumference, was recorded for analysis.

Ascending aortic length (L, cm) was defined as the centerline distance from the aortic annulus to the origin of the innominate artery (Figure 1A,B). Maximum ascending aortic diameter (D, cm) was defined as the largest measurement obtained from cross-sectional CTA images at the aortic root, sinotubular junction (STJ), and tubular ascending aorta. At the aortic root level, CT-based measurements were performed using sinus-to-sinus dimensions. At the STJ and tubular ascending aorta, diameters were measured in planes perpendicular to the vessel centerline. In asymmetric or oval cross-sections, the longest diameter and its perpendicular short-axis diameter were identified in the measurement plane. The maximal diameter, measured as the distance between two oppositely positioned points along the circumference, was recorded for analysis (Figure 1C).

Body surface area (BSA, m^2^) was calculated using the Du Bois formula [17], and patient height was recorded at clinical admission. Derived indexed parameters were calculated as follows: the aortic size index (ASI, cm/m^2^) was defined as the ratio of maximum ascending aortic diameter to BSA [15]; the aortic height index (AHI, cm/m) was defined as the sum of ascending aortic diameter and length divided by patient height [5,15]; the aortic length index (ALI, cm/m^2^) was defined as the ratio of ascending aortic length to BSA. Cross-sectional aortic area (CSA) was calculated using the formula πD^2^/4, which was then indexed to height (CSA/H) by dividing by patient height [18]. This calculation, in line with standard practice, assumed a near circular cross-sectional geometry.

### 2.6. Estimated Pre-Dissection Aortic Dimensions

To account for acute dissection-related geometric distortion, we also calculated estimated pre-dissection aortic dimensions. Estimated pre-event AsA diameter was derived by reducing the measured AsA diameter by 18%, in accordance with previously reported correction model [5]. Estimated pre-event AsA length was calculated by reducing the measured AsA length by 2.7%, following prior observations demonstrating minimal elongation during acute dissection [5]. These correction factors were applied for exploratory purposes only and were intended to provide an approximate representation of pre-dissection geometry rather than clinically actionable estimates.

### 2.7. Data Analyses

Statistical analyses were performed using GraphPad Prism 10 (GraphPad Software, La Jolla, CA, USA) and JMP Pro 17 (SAS Institute Inc., Cary, NC, USA), with a two-tailed significance threshold of *p* < 0.05. Normality was assessed using the Shapiro–Wilk test, and results are reported as mean (standard deviation; SD) for normally distributed variables or as median (interquartile range; IQR) for non-normally distributed variables. Frequency distribution graphs, including histograms with kernel density curves and grouped bar charts, were constructed in GraphPad Prism 10 using the Freedman–Diaconis bin-width rule to characterise the distribution of key variables across the cohort.

Between-group differences in continuous variables were assessed using the independent samples *t*-test when normal distribution was observed, or the Mann–Whitney U test in non-normal distributions. Categorical variables were compared using the chi-squared test or Fisher’s exact test, as appropriate. Pairwise correlations among all continuous anthropometric and morphometric variables were assessed using Spearman’s rank correlation matrix, visualised as a colour-coded heatmap generated in JMP Pro 17 to identify natural variable groupings. To evaluate the reproducibility of morphometric measurements, inter-rater reliability was assessed for the main CTA-derived aortic measurements, namely maximum ascending aortic diameter and ascending aortic length. Agreement between raters was quantified using the intraclass correlation coefficient (ICC), calculated using a two-way mixed-effects model with absolute agreement, and reported alongside 95% confidence intervals (95% CI). ICC values between 0.75 and 0.90 were considered to reflect good reliability [19].

## 3. Results

In our cohort of 65 patients, the mean age of the overall cohort was 61 years (SD 13, range 31 to 86), with 35 (54%) patients aged < 65 years while 30 (46%) patients were aged ≥ 65 years. The cohort was predominantly male (*n* = 45; 69%). Age differed between sexes, with women being older than men (*p* = 0.01)—the mean age of females was 67 (SD 11, range 48 to 85) years compared with the mean age of males (mean: 58 years, SD 13, range 31 to 86). A bicuspid aortic valve was identified in only four (6.2%) patients, while the remaining patients had a tricuspid aortic valve. Coronary artery involvement was observed in 4.6% of cases. Extension of the dissection into the brachiocephalic vessels occurred in 76.9% of patients. Isolated involvement of the AsA was present in 15.4% of cases, whereas further propagation of the dissection into the downstream aorta occurred in 84.6%. A history of hypertension was documented in 53 patients (81.5%).

### 3.1. Inter-Rater Reliability

Inter-rater reliability (ICC) testing of CTA-based morphometric measurements showed good concordance for maximum ascending aortic diameter (estimate: 0.86, 95% CI: 0.80 to 0.90), with excellent concordance observed for ascending aortic length (estimate: 0.92, 95% CI: 0.88 to 0.95).

### 3.2. Distribution of Morphometric Parameters Across Age Groups

At the time of acute dissection, an AsA diameter ≥ 5.5 cm was present in 34.3% (12/35) of patients aged < 65 years and 36.7% (11/30) of those aged ≥ 65 years. By comparison, an AsA length > 11.5 cm was observed more frequently, in 51.4% (18/35) and 63.3% (19/30) of patients, respectively. Nonetheless, these distributional differences remained not significant for both aortic diameter and length across the age groups (Table 1). Patients < 65 years were taller and had greater BSA than patients ≥ 65 years (*p* = 0.001 and *p* = 0.013, respectively). Despite smaller body size, patients ≥ 65 years demonstrated higher indexed parameters, including ALI (*p* = 0.004), AHI (*p* = 0.015), and ASI (*p* = 0.059).

### 3.3. Distribution of Morphometric Parameters Across Sex

A CTA-measured AsA diameter ≥ 5.5 cm was observed in 40.0% of women (8/20) and 33.3% of men (15/45). In contrast, a CTA-measured AsA length > 11.5 cm was more common in men than in women, occurring in 64.4% (29/45) and 40.0% (8/20), respectively. Aortic diameter and length also did not differ between sexes (Table 2). Likewise, CSA and CSA/H did not differ significantly between sexes. Men had greater height and BSA (both *p* < 0.001), whereas women showed higher indexed parameters: ALI, AHI and ASI (*p* < 0.001, *p* = 0.045 and *p* < 0.001, respectively).

### 3.4. Distribution of ASI Across Age Groups and Sex

**Figure 2:**
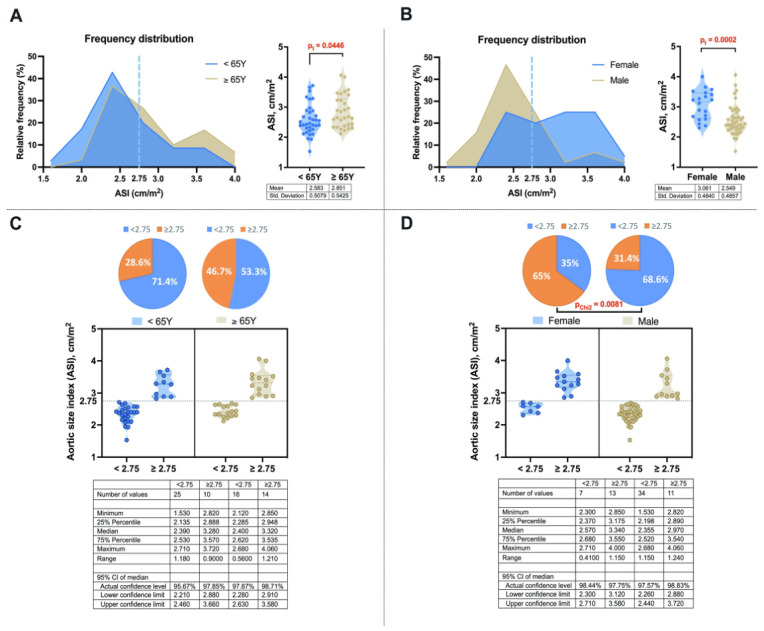
Distribution of computed tomography angiography (CTA) measured aortic size index (ASI) across age and sex groups. (**A**) Relative frequency distribution of ASI across age groups (<65 years and ≥65 years), shown as histograms with overlaid density curves, accompanied by violin plots with individual data points (dots) and between-group comparison. (**B**) Relative frequency distribution of ASI across sex groups (female and male), shown as histograms with overlaid density curves, accompanied by violin plots with individual data points and between-group comparison. (**C**) Distribution of ASI according to the 2.75 cm/m^2^ threshold across age groups, shown as relative frequency pie charts, violin plots with individual data points, a threshold reference line, and summary statistics. (**D**) Distribution of ASI according to the 2.75 cm/m^2^ threshold across sex groups, shown as relative frequency pie charts, violin plots with individual data points, a threshold reference line, and summary statistics. Summary statistics indicate the number of patients, median with IQR, range, and 95% confidence intervals of the median. The dotted lines illustrate the ASI ≥ 2.75 cm/m^2^ threshold used for group stratification. p_t_—significance level according to the independent samples *t*-test; p_Chi2_—significance level according to the chi-squared test.

The distribution of patients across ASI-defined groups (<2.75 cm/m^2^ and ≥2.75 cm/m^2^) showed significant differences when stratified by sex, but no differences when stratified by age groups (Figure 2). Patients with ASI < 2.75 cm/m^2^ had greater height and BSA (*p* = 0.004 and *p* < 0.001), while patients with ASI ≥ 2.75 cm/m^2^ had higher ALI ratio and AHI (both *p* < 0.001).

### 3.5. Distribution of Estimated Pre-Dissection Aortic Dimensions

The estimated pre-dissection maximal AsA diameter exceeded 5.5 cm in three patients in each age group (8.6% of those aged < 65 years and 10% of those aged ≥ 65 years). Among patients < 65 years, an estimated pre-dissection AsA length exceeded 11.5 cm in 17 cases (48.6%), and in 16 patients in the ≥65 years group (53.3%). Based on sex, estimated pre-dissection maximal AsA diameter exceeded 5.5 cm in only one woman (5%) and five men (11.1%). Similarly, estimated pre-dissection AsA length exceeded 11.5 cm in six women (30%) and twenty-nine men (64.4%).

**Figure 3:**
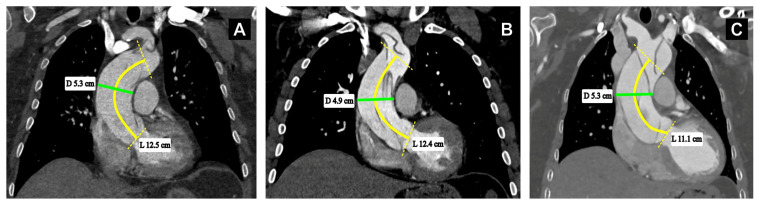
Representative computed tomography angiography (CTA) images highlighting differences in ascending aortic (AsA) length (L) and maximal diameter (D). (**A**) Representative image of ascending aortic elongation, with an AsA L of 12.5 cm and a maximal AsA D of 5.3 cm. (**B**) Representative image of pronounced ascending aortic elongation, with an AsA L of 12.4 cm and a maximal AsA D of 4.9 cm. (**C**) Representative image with an AsA L of 11.1 cm and a maximal AsA D of 5.3 cm. In all panels, the curved yellow line indicates the aortic centerline used for length measurement, the green line labelled D indicates the maximal ascending aortic diameter. The yellow dashed oblique lines indicate the proximal and distal reference landmarks. Ascending aortic length was measured along the centerline from the aortic annulus to the origin of the innominate artery, and diameter was measured in a plane perpendicular to the vessel centerline.

No significant differences in estimated pre-dissection aortic diameter and length were observed between age groups (Table 1). CSA and CSA/H also did not differ. However, indexed parameters such as ALI, AHI, and ASI were greater in patients ≥ 65 years. Between sexes, no differences in estimated aorta diameter, length or CSA were observed (Table 2). Women demonstrated higher indexed parameters, with greater ALI, AHI and ASI than men. After stratification according to ASI cutoff, the estimated diameter was greater in the higher-ASI group (*p* < 0.001), whereas estimated length did not differ. Patients with higher ASI also had higher ALI, AHI, and ASI (all *p* < 0.001), and greater CSA and CSA/H (both *p* < 0.001). Our illustrative images further support our observations, in that ascending aortic elongation may be observed at different maximal diameters, including below the conventional operative threshold, and that similar maximal diameters may be present both with and without elongation (Figure 3).

### 3.6. Correlation Analysis and Clustering

**Figure 4:**
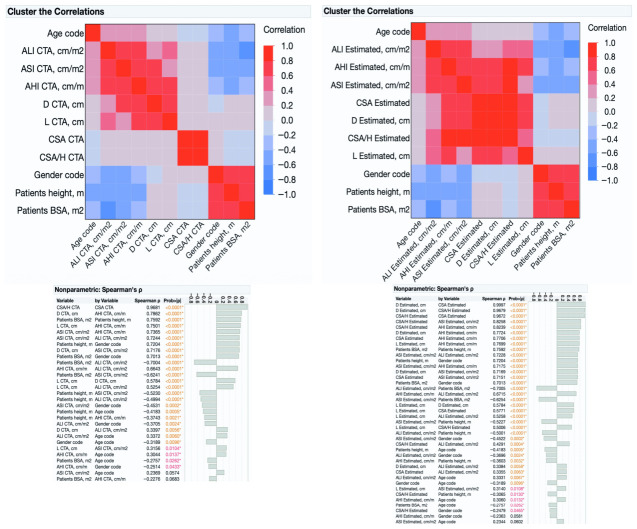
Correlation matrices of the main aortic and body-size parameters for computed tomography angiography (CTA) measured values at presentation and estimated pre-dissection values. The heatmaps illustrate the direction and strength of pairwise correlations according to the color scale from blue (negative correlation) to red (positive correlation). The accompanying tables present the corresponding Spearman correlation coefficients (ρ) and *p* values for significant (*) associations. *p* = 0.05–0.01 marked with red colour, *p* < 0.01—marked with orange colour.

Similar correlation patterns were observed for both CTA-measured and estimated parameters (Figure 4). The strongest positive correlations were found between CSA/H and CSA, as well as between BSA and height. Strong positive correlations were observed between aortic diameter and length and among indexed parameters (AHI, ALI and ASI), whereas ALI showed a negative correlation with BSA in both groups. In the estimated parameters, additional positive correlations were observed between CSA/H and ASI, CSA/H and AHI, as well as between CSA and AHI and CSA and ASI.

## 4. Discussion

In this nationwide cohort, we observed surgically treated ATAAD almost twice more frequently in women as in men, consistent with the male predominance reported in previous studies [9]. A substantial proportion of patients across age and sex subgroups presented below the conventional 5.5 cm diameter threshold recommended in earlier guidelines [20]. At presentation, only approximately one third of patients had a CTA-measured ascending aortic diameter exceeding 5.5 cm, consistent with previously reported IRAD registry observations that many dissections occur below conventional operative criteria [4]. These findings support the viewpoint that reliance on a single diameter cutoff may not fully capture the spectrum of patients presenting with acute dissection [15,21]. Perez et al., likewise reported that the diameter at the time of type A dissection was often close to the 5.0 cm range, although their measurement methodology differed from that used in the present study [8].

In our cohort, a considerable proportion of patients had diameters below 5.0 cm at presentation, and this proportion increased further after estimation of pre-dissection dimensions. Imaging studies comparing pre- and post-dissection measurements have shown that the ascending aorta enlarges acutely at the time of dissection, resulting in larger diameters on post-dissection CTAs [22]. Modelling and reconstruction approaches have similarly suggested that the true pre-dissection diameter is substantially smaller than that measured during the acute event [6,23]. For example, Tozzi et al., reported that 87.7% of patients with type A aortic dissection had a modelled pre-dissection maximum diameter below 45 mm (or 4.5 cm) [6], while Koechlin et al., found that the mean ascending aortic diameter decreased from 46 mm at dissection to a modelled pre-dissection diameter of 32.3 mm [23]. Our sex-stratified analysis further demonstrated that only a small minority of patients in either sex remained above the 5.5 cm threshold after estimation of pre-dissection size, which is consistent with these previous reports indicating that post-dissection measurements may substantially overestimate pre-event aortic size.

Collectively, these findings indicate that many patients with ATAAD present below conventional diameter thresholds at the time of dissection. Nonetheless, the present results should not be interpreted as definitive evidence to redefine prophylactic surgical thresholds for stable aneurysms, but rather as a supporting trend for further investigation of complementary morphometric risk markers. In our cohort, ascending aortic length measurements exceeded 11.5 cm more frequently than diameter-based thresholds, both at presentation and after correction. Elongation beyond this threshold was observed in a substantial proportion of both younger and older patients, without significant differences in absolute length between age groups or sexes. Previous CTA-based studies have demonstrated that patients with acute type A dissection had longer ascending aortas than controls, indicating that aortic length may be complementary to diameter [9,24].

The frequent observation of ascending aortic elongation in our cohort may reflect underlying biomechanical mechanisms of aortic remodeling. Radial dilatation is primarily governed by circumferential wall stress proportional to pressure and vessel radius [25], whereas aortic geometry influences local stress distribution [26]. Elongation may therefore reflect cumulative longitudinal forces related to cardiac motion and aortic tethering, mechanisms implicated in dissection initiation and propagation. In this context, a complementary biomechanical hypothesis is that acute type A dissection may result not only from diameter-related circumferential wall stress but also from longitudinal wall failure promoted by chronic aortic elongation and cyclic longitudinal strain [27,28]. This interpretation aligns with observations that geometric factors beyond diameter, including parameters related to aortic length and curvature, contribute to dissection susceptibility [29,30]. From a practical perspective, these observations suggest that assessment of ascending aortic length, in addition to diameter, may improve morphometric evaluation during diagnostic CTA and may provide complementary information for postoperative follow-up aimed at detecting progressive geometric remodeling.

We also observed that sex-related patterns in aortic geometry were more evident in relative, but not absolute, diameter enlargement than in longitudinal elongation. For contextual interpretation, our median estimated pre-dissection values were compared with the normal ascending aortic dimensions reported in the control group described by another similar study [16]. In that control group, women had smaller median ascending aortic diameters than men (31.7 mm vs. 36.1 mm) and shorter median lengths (79.6 mm vs. 88.0 mm). In contrast, in our cohort, median estimated diameters were nearly identical between sexes (women 4.31 cm vs. men 4.35 cm), corresponding to a greater relative increase in diameter in women compared with men when referenced with the reported normal medians (+37% vs. +19%). Conversely, when compared with the normal reference values reported by Eliathamby et al., median ascending aortic length demonstrated a comparable proportional increase in both sexes (women: 7.96 to 10.9 cm, +37%; men: 8.8 to 12.16 cm, +38%) [16]. In our cohort, the female-to-male ratio of estimated median length (0.90) closely paralleled the female-to-male height ratio (0.91), suggesting proportionality between longitudinal aortic dimensions and body height at the group level.

Together, these observations indicate that sex-related differences in pre-dissection aortic geometry are predominantly related to transverse expansion rather than disproportionate longitudinal elongation. This interpretation is consistent with previous observations that women with acute type A aortic dissection may present with relatively greater ascending aortic dilatation than men [3]. Beyond absolute dimensions, body-size-indexed parameters demonstrated systematic demographic variation. Older patients exhibited higher ALI, AHI and ASI ratios despite having smaller height and body surface area, while absolute aortic diameter and length did not differ between age groups. This suggests that age-related differences become more apparent when aortic dimensions are interpreted relative to body size, with anthropometric variables serving primarily to contextualize indexed aortic measures rather than acting as independent clinically informative markers. Similar findings have been reported previously, suggesting that body-size indexing may influence the interpretation of aortic dimensions and risk assessment [31].

When indexed to body size, sex-related differences became more apparent. Although absolute ascending aortic diameter, length and cross-sectional area did not differ between sexes, women demonstrated higher indexed parameters, whereas men had larger anthropometric measurements. These findings suggest that reliance on absolute diameter thresholds may underestimate proportional aortic enlargement in women. Previous thoracic aortic aneurysm cohorts have reported similar sex-related disparities and worse outcomes in women [32], while ASI has been shown to improve risk discrimination compared with diameter alone [33]. However, because the present study did not include outcome-based validation, indexed parameters such as ALI, AHI, and ASI should be regarded here as descriptive and hypothesis-generating measures rather than as established predictors of adverse clinical events. Consistent with these observations, the strong correlations observed among the indexed parameters (AHI, ALI and ASI) indicate that different indexing approaches capture closely related aspects of relative aortic size.

This finding is in line with previous work showing that indexing aortic dimensions to body size improves the interpretation of aortic dilatation by accounting for anthropometric variability [5]. In contrast, body-size variables (height and BSA) formed a separate cluster and showed limited association with absolute aortic size measures, suggesting that variation in aortic dimensions in this cohort cannot be explained solely by differences in body size. The negative correlation between ALI and BSA further indicates that relative aortic length decreases with increasing body size, supporting the concept that aortic elongation represents a distinct component of aortic remodeling rather than a simple scaling effect [9]. Importantly, the preservation of similar correlation patterns between CTA-measured and estimated parameters suggests that the estimation approach maintained the internal relationships among aortic size metrics. Stratification according to ASI reinforced these relationships. Patients exceeding the 2.75 cm/m^2^ threshold exhibited larger absolute diameters and higher ALI ratios and AHI, consistent with more advanced size-normalized remodeling. Similar patterns in estimated pre-dissection ASI and AHI argue against these differences being solely attributable to acute geometric distortion [34].

While echocardiography, particularly transthoracic and trans-esophageal echocardiography, remains an essential diagnostic modality in acute type A aortic dissection, CTA remains the preferred imaging modality for detailed morphometric assessment, as it provides more reproducible evaluation of aortic diameter, length, branch vessel involvement, and the overall extent of dissection [35,36,37,38]. In this context, CTA enabled standardized assessment of predefined morphometric parameters, including aortic diameter, length, cross-sectional area, and indexed measurements.

### Limitations and Future Directions

The present study has several limitations. Cross-sectional aortic area was derived from a single maximal diameter assuming circular geometry and therefore did not account for potential elliptical cross-sectional morphology or localized geometric variation. In addition, multi-axis diameter measurements, perimeter-based area assessment, and sequential cross-sectional analysis were not performed. These approaches may provide a more detailed characterization of regional aortic geometry and should be considered in future studies. Localized luminal irregularities or focal narrowing were not specifically analyzed, as the study focused on global morphometric parameters of the ascending aorta.

Furthermore, geometric features beyond the predefined parameters, as well as biological determinants of wall vulnerability, were not captured by the current retrospective design. Future studies incorporating true pre-event imaging are required to better characterize individual variability in aortic geometry prior to dissection. In addition, further investigation of geometric descriptors such as aortic curvature may provide complementary insights into aortic morphology and biomechanical stress distribution. Assessment of aortic wall histopathology, microstructural integrity, and patient-specific biomechanical properties may also provide important information beyond geometric measurements alone. Although the present study used three-dimensional centerline reconstructions to assess aortic length and centerline-perpendicular diameters, formal curvature analysis was not performed. Such analysis would require additional geometric modelling and dedicated computational methods, which were beyond the scope of this retrospective study.

Next, important physiological variables, including systolic blood pressure, heart rate, and ECG-derived functional parameters, were not consistently available in a standardized form across the cohort and therefore could not be incorporated into the present analysis. Future prospective studies integrating CT-based morphometric evaluation with hemodynamic and ECG-derived functional parameters may provide a more comprehensive characterization of dissection susceptibility. In addition, the correction factors used to estimate pre-dissection diameter and length were derived from previously published cohorts and were not independently validated in the present population. Finally, the study cohort included only surgically treated ATAAD patients and therefore excluded patients who died before hospital admission, were not referred for surgery, or were deemed unsuitable for operative treatment. As a result, the study population may not fully represent the entire ATAAD spectrum, and the findings should be interpreted primarily within the context of surgically treated patients. Furthermore, this resulted in a relatively small cohort, and the subgroup analyses presented may not have had sufficient statistical power to detect meaningful differences.

## 5. Conclusions

In this retrospective nationwide cohort, a substantial proportion of patients with acute type A aortic dissections presented with ascending aortic diameters below conventional surgical thresholds, and estimation of pre-dissection dimensions further increased the proportion below these thresholds. Ascending aortic elongation was frequently observed, suggesting that longitudinal aortic geometry may represent an additional feature of aortic remodeling. Indexed morphometric parameters (ALI, AHI, and ASI) revealed differences in relative aortic size that were not apparent from absolute measurements alone. Together, these observations suggest that CT-based morphometric assessment incorporating indexed parameters and ascending aortic length may provide complementary structural information beyond diameter measurements alone. However, given the retrospective design, limited sample size, absence of control subjects, and reliance on estimated pre-dissection dimensions, these findings need further validation in larger prospective multicenter cohorts.

## Figures and Tables

**Table 1:** Overview of morphometric measurements derived from the computed tomography angiography (CTA) at the time of dissection and estimated values at pre-dissection, stratified by age groups.

Measurement *	At Dissection			Estimated Pre-Dissection		
	<65 Years (*n* = 35)	≥65 Years (*n* = 30)	*p* Value	<65 Years (*n* = 35)	≥65 Years (*n* = 30)	*p* Value
AsA diameter (D; cm)	5.10 (4.85 to 5.70)	5.30 (4.93 to 5.7)	0.452	4.18 (3.98 to 4.68)	4.35 (4.04 to 4.67)	0.489
AsA length (L; cm)	11.70 (11.00 to 12.55)	12.05 (11.12 to 12.9)	0.288 ‡	11.38 (10.70 to 12.21)	11.72 (10.83 to 12.55)	0.287 ‡
AsA length/diameter Ratio	2.18 (1.97 to 2.34)	2.16 (1.99 to 2.88)	0.990 ‡	2.66 (2.40 to 2.85)	2.63 (2.43 to 2.78)	0.999 ‡
Aortic height index (AHI; cm/m)	9.73 (9.09 to 10.29)	10.35 (9.72 to 10.72)	0.015	8.99 (8.41 to 9.54)	9.57 (9.03 to 9.90)	0.015
Aortic length index (ALI; cm/m^2^)	5.67 (5.13 to 6.04)	6.12 (5.64 to 6.75)	0.004 ‡	5.52 (4.99 to 5.88)	5.96 (5.49 to 6.57)	0.004 ‡
Aortic size index (ASI; cm/m^2^)	2.46 (2.28 to 2.85)	2.66 (2.39 to 3.23)	0.059	2.02 (1.87 to 2.34)	2.18 (1.95 to 2.65)	0.057
Cross-sectional aortic area (CSA; cm^2^)	22.06 (18.10 to 25.97)	20.83 (18.86 to 24.41)	0.984	13.72 (12.44 to 17.17)	14.86 (12.82 to 17.15)	0.489
CSA/height index (cm^2^/m)	13.27 (10.60 to 15.71)	11.90 (10.67 to 13.91)	0.426	7.86 (6.87 to 9.87)	8.77 (7.56 to 10.63)	0.216
Body surface area (BSA; m^2^)	2.09 (2.00 to 2.23)	1.99 (1.83 to 2.13)	0.013 ‡	-	-	-
Patient height (m)	1.80 (1.73 to 1.83)	1.70 (1.61 to 1.76)	0.001 ‡	-	-	-

* Values are presented as median and IQR (Q1 to Q3). ‡ *p* value derived from independent samples *t*-test. Elsewhere *p* value derived from Mann–Whitney U test.

**Table 2:** Overview of morphometric measurements derived from the computed tomography angiography (CTA) at the time of dissection and estimated values at pre-dissection, stratified by sex.

Measurement *	At Dissection			Estimated Pre-Dissection		
	Male (*n* = 45)	Female (*n* = 20)	*p* Value	Male (*n* = 45)	Female (*n* = 20)	*p* Value
AsA diameter (D; cm)	5.10 (4.80 to 5.60)	5.30 (4.97 to 5.80)	0.442	4.18 (3.94 to 4.59)	4.35 (4.08 to 4.76)	0.438
AsA length (L; cm)	12.20 (11.00 to 12.90)	11.20 (11.02 to 11.85)	0.098 ‡	11.87 (10.7 to 12.55)	10.90 (10.73 to 11.53)	0.100 ‡
AsA length/diameter Ratio	2.19 (2.09 to 2.33)	2.02 (1.91 to 2.19)	0.037 ‡	2.68 (2.54 to 2.84)	2.46 (2.33 to 2.67)	0.037 ‡
Aortic height index (AHI; cm/m)	9.78 (9.12 to 10.46)	10.24 (9.88 to 10.73)	0.045	9.06 (8.45 to 9.71)	9.44 (9.17 to 9.93)	0.060
Aortic length index (ALI; cm/m^2^)	5.80 (5.11 to 6.15)	6.30 (5.70 to 7.18)	<0.001 ‡	5.64 (4.98 to 5.98)	6.13 (5.54 to 6.98)	<0.001 ‡
Aortic size index (ASI; cm/m^2^)	2.42 (2.26 to 2.68)	3.17 (2.66 to 3.43)	<0.001	1.98 (1.86 to 2.20)	2.60 (2.17 to 2.81)	<0.001
Cross-sectional aortic area (CSA; cm^2^)	22.06 (18.86 to 24.63)	20.44 (17.91 to 29.22)	1.000	13.72 (12.19 to 16.55)	14.86 (13.08 to 17.78)	0.438
CSA/height index (cm^2^/m)	12.75 (10.97 to 14.84)	12.39 (10.00 to 16.7)	0.915	7.86 (6.89 to 9.40)	9.15 (8.55 to 10.85)	0.049
Body surface area (BSA; m^2^)	2.13 (2.02 to 2.23)	1.85 (1.77 to 1.94)	<0.001	-	-	-
Patient height (m)	1.79 (1.75 to 1.83)	1.63 (1.59 to 1.67)	<0.001 ‡	-	-	-

* Values are presented as median and IQR (Q1 to Q3). ‡ *p* value derived from independent samples *t*-test. Elsewhere *p* value derived from Mann–Whitney U test.

## Data Availability

All data used in this article are included in the article. Additional data and materials are available upon request from the corresponding authors.

## Abbreviations

The following abbreviations are used in this manuscript:

AsA	Ascending aorta
AsAA	Ascending aortic aneurysm
ATAAD	Acute Stanford type A aortic dissection
ASI	Aortic size index
CTA	Computed tomography angiography
D	Aortic diameter
L	Aortic length
BSA	Body surface area
AHI	Aortic height index
ALI	Aortic length index
CSA	Cross-sectional aortic area
CSA/H	Cross-sectional aortic area indexed to height
